# Combined effects of the rs9810888 polymorphism in calcium voltage-gated channel subunit alpha1 D (*CACNA1D*) and lifestyle behaviors on blood pressure level among Chinese children

**DOI:** 10.1371/journal.pone.0216950

**Published:** 2019-05-30

**Authors:** Yi-de Yang, Jie-Yun Song, Shuo Wang, Yang Wang, Qi-Ying Song, Yan-hui Dong, Chen-Xiong Li, Hai-Jun Wang, Jun Ma

**Affiliations:** 1 Institute of Child and Adolescent Health, School of Public Health, Peking University Health Science Center, Beijing, China; 2 Key Laboratory of Molecular Epidemiology of Hunan Province/Teaching and Researching Office of Child and Adolescent Health, School of Medicine, Hunan Normal University, Changsha, China; 3 Department of Maternal and Child Health, Peking University Health Science Center, Beijing, China; Sao Paulo State University (UNESP), BRAZIL

## Abstract

**Backgrounds:**

A recent GWAS Study found a new locus (rs9810888 in *CACNA1D*) was associated with blood pressure (BP) in Chinese adults. But whether the association exists in children is unknown. Whether lifestyle behaviors could interact with rs9810888 on BP is not clear. This study aimed to identify the association between rs9810888 and BP in children, and also explore the gene-lifestyle interaction.

**Methods:**

A case-control study was conducted among 2030 Chinese children aged 7 to 18 years. Genotyping was conducted by using the matrix-assisted laser desorption/ionization time-of-flight mass spectrometry. Lifestyle behaviors were investigated with questionnaire.

**Results:**

With adjustment for age, age square, sex, study group and body mass index (BMI), rs9810888 was significantly associated with diastolic BP (DBP) (b = 1.69, *p* = 0.021) and mean arterial BP (MAP) (b = 1.56, *p* = 0.010). Stratified analysis showed that the rs9810888 GG genotype carriers had higher DBP than GT/TT carriers (b = 3.78, *p* = 0.023) in the subgroup having protein intake (meat/fish/soybeans/egg) <twice/day, but not in the subgroup ≥twice/day. In addition, in the subgroup with screen time≥2h/day, the rs9810888 GG genotype carriers had higher DBP than GT/TT carriers (b = 5.49, *p* = 0.012), but not in the subgroup with screen time<2h/day. For MAP, in the subgroup having either fruits or vegetables < twice/day, the rs9810888 GG genotype carriers had higher MAP than GT/TT carriers (b = 2.64, *p* = 0.037), but not in the subgroup with both fruits and vegetables intake ≥twice/day. Additionally, in the subgroup with screen time≥2h/day, the rs9810888 GG genotype carriers had higher MAP than GT/TT carriers (b = 4.80, *p* = 0.017), but not in those with screen time<2h/day.

**Conclusions:**

The *CACNA1D* rs9810888 polymorphism was significantly associated with DBP and MAP. In addition, unhealthy lifestyle behaviors and rs9810888 GG genotype had combined effects on BP level among Chinese children.

## Backgrounds

Hypertension is the largest metabolic contributor to global disability-adjusted life-years of both men and women in 2016, which is a major public health problem all over the world[1]. Blood pressure (BP) is known as a complex phenotype affected by genetic and environmental factors. Genetically, the heredity of BP is estimated to be about 40~60%[2, 3]. But only a small part (2~3%) of the BP variance could be explained by the polymorphisms identified in Genome-Wide Association Study (GWAS)[4].

Environmental factors also play an important role in the etiology of hypertension. In addition, environmental risk factor could be modified, which mainly include physical activity, sedentary behaviors, and dietary behaviors[5].Thus, the gene-environmental interaction would be useful for developing practical prevention measures. Personalized lifestyle recommendation based on genetic background are being promoted in public health and precision medicine in recent years[6].

A recent GWAS found a polymorphism (rs9810888) in calcium voltage-gated channel subunit alpha1 D gene (*CACNA1D)* was associated with BP in Chinese adults[7]. *CACNA1D* is a member of the α-1 subunit family of voltage-dependent calcium channels, most of which play an important role in BP regulation and are targets of antihypertensive drugs calcium channel blockers. *CACNA1D*, together with *CACNA1C*, are widely expressed in (neuro)endocrine cells and electrically excitable cells of the cardiovascular system, especially vascular smooth muscle and cardiac muscle cells[8]. The polymorphisms of these genes could lead to channel activation at more hyperpolarized membrane potentials, resulting in increased Ca^2+^ influx in the pathogenesis of disease[9]. But whether the newly identified locus rs9810888 in *CACNA1D* was associated with BP in children is unknown. Therefore, we hypothesize that rs9810888 polymorphism is associated with BP in Chinese children, then the primary objective of the study was to explore the association in Chinese children.

Besides, gene-lifestyle interaction is now considered as an important explanation of high blood pressure[10]. Many studies have illustrated the gene-lifestyle interaction in the level of blood pressure or risk of hypertension[11, 12]. Recently, Xi’s study found the calcium channel gene *ATP2B1*’s polymorphism could interact with lifestyle factors to have an effect on BP among children[11]. However, whether lifestyle behaviors could interact with rs9810888 on BP phenotypes has not been explored or reported before. We hypothesize that rs9810888 polymorphism may have interactive effect with lifestyle behaviors on BP in children, so the second objective of the study was to explore the gene-lifestyle interaction between the *CACNA1D* rs9810888 polymorphism and lifestyle behaviors on BP.

## Methods

### Participants

A case-control study was conducted among 2030 children aged 7 to 18 years old from two independent study groups in Beijing, China. The first study group came from the study on Adolescent Lipids, Insulin Resistance, and candidate genes (ALIR) in nine middle schools of Dongcheng District of Beijing. The second study was from Comprehensive Prevention project for Overweight and Obese Adolescents (CPOOA) in five elementary and middle schools of Haidian District of Beijing. The sampling strategies for the two study groups were described in detail in the previous studies[13–15].The ALIR study recruited 937 children aged 14 to 17 years old. The CPOOA study recruited 1093 children aged 7 to 18 years old[14]. In the two study groups, nine participants were excluded because of absence of BP phenotype data, so totally, 2021 children were included in the current study.

### Ethics approval and consent to participate

Both the ALIR study and CPOOA study were approved by the Ethics Committee of Peking University Health Science Center. There are two numbers of the approval of ethic committee, namely IRB00001052-06082 for the ALIR study and IRB00001052-06084 for the COOPA study. Written informed consent was provided by all participants and, in the case of minors, their parents. Studies were performed according to the Declaration of Helsinki.

### Measurements

Height, weight and BP were measured by standard protocols, which is described in detail previously[16]. BP was calculated by averaging three measurements at one visit. It was measured three times with 5-minute time interval. BP was measured according to the recommendation of the National High Blood Pressure Education Program Working Group for Children and Adolescents[17]. Systolic BP (SBP) was defined as the onset of “tapping” Korotkoff sound (K1), and diastolic BP (DBP) was defined as the fifth Korotkoff sound (K5). Mean arterial pressure (MAP) and pulse pressure (PP) was calculated as follow: MAP = (SBP+2×DBP)/2 (mmHg), PP = SBP-DBP (mmHg)[18]. Systolic high blood pressure(SHBP) and diastolic high blood pressure (DHBP) were defined as SBP or DBP ≥ the age- and sex-specific 95th percentile of a representative Chinese children population, respectively[19]. HBP was defined as SHBP and/or DHBP.

### Lifestyle behaviors

Lifestyle behaviors included dietary behaviors, physical activity and sedentary behaviors were measured by questionnaire. Dietary behaviors, including consumption of fruits, vegetables, high calorie foods (fried chips/cakes/cookies), protein intake (meat/fish/soy beans/egg), western food and soft drink were measured in the questionnaire. The frequencies of eating fruit, vegetable, high calorie foods (fried chips/cakes/cookies), soft drink were investigated with the options of “Never”, “1~3 times”, “4~6 times”, “daily”, “twice per day”, “3 times per day”, or “more than 3 times per day”. Physical activity and screen time were also measured by a questionnaire[14]. Time spent on physical activity each day was measured with the options of “Never”, “0~0.5 hour per day”, “0.5~1 hour per day”, “1~2 hours per day”, “2~3 hours per day”, “3~4 hours per day”, and “more than 4 hours per day”. The dietary behavior and physical activity variables were classified into two categories based on the national recommendation of nutrition and physical activity for Chinese children[20]. For sedentary behaviors, we investigated the screen time. Screen time included time spent on the television/video viewing and computer/video game playing, and were categorized into < 2 hours/day or ≥2 hours /day according to the American Academy of Pediatrics [21].

### Genotyping

Genomic DNAs of participants were isolated from blood leukocytes by the phenol-chloroform extraction method. The *CACNA1D* rs9810888 polymorphism was assayed by using the matrix-assisted laser desorption/ionization time-of-flight mass spectrometry (MALDI-TOF MS, Agena). All the experiments were conducted by investigators who were blind to the phenotypes. The genotyping call rates of rs9810888 was 99.95%.

### Statistical analyses

The Hardy-Weinberg equilibrium was tested with the Chi-square test for the genotype data of the control group (non-HBP group). The quantitative variables were described as mean and standard deviations (SD). The categorical variables were described as percentages. Differences between HBP group and non-HBP group were compared with Chi-square tests (categorical variables) and t-tests (quantitative variables). The effect of rs9810888 on BP phenotypes (quantitative variables) and interaction terms (genotype × lifestyle variables) were estimated with multivariate general linear model with age, age square, sex and BMI as covariates. Multivariate logistic regression model with age, age square, sex and BMI as covariates was conducted to test the significance of interaction terms (genotype × lifestyle variables) on risk of HBP. All analysis was conducted under a recessive genetic model (GG = 1, GT/TT = 0). For the genetic model selection, we calculated Akaike information criteria (AIC) and Bayesian information criteria (BIC), which are both useful in model comparison[22, 23], i.e. the smaller AIC and BIC were, the better the corresponding model fitted to the data. In our study, rs9810888 was significantly associated with DBP under a recessive model, AIC and BIC values all indicated the recessive genetic model are the optimal model after model comparison (**S1 Table**). The statistical analyses were performed with SPSS for Windows (version 20.0, SPSS Inc., Chicago, IL, USA). In the present study, we adjusted covariates of age, age square, sex and BMI as the GWAS study which identified this new locus. Considering age and age square may have a great weight to the analysis, we did sensitivity analysis to only adjust age without adjustment of age square.

## Results

### The general characteristics of the study population

In the present study, a total of 2021 children and adolescents were involved. The general characteristics of the participants were showed in **Table 1**. Mean age of the participants were 12.9 years old, 60.1% of them were boys, average BMI, SBP, DBP, MAP and PP were 23.8 kg/m^2^, 113.5 mmHg, 62.2 mmHg, 79.3 mmHg and 51.2 mmHg, respectively. The participants with HBP had significantly higher age, BMI, SBP, DBP, MAP and PP than the non-HBP participants (all *p values*<0.001). The frequency of TT, GT and GG genotype was 36.1%, 48.1% and 15.8%, respectively. The genotype distribution of *CACNA1D* polymorphism rs9810888 in the control group was in Hardy-Weinberg Equilibrium (*p* = 0.448), while the G allele frequency in the control group was 39.7%.

**Table 1:** General characteristics and behavior characteristics of the study population.

Variables		Total(N = 2021)	Non HBP(n = 1319)	HBP(n = 702)	p*
Sex	boys	1215(60.1%)	724(54.9%)	491(69.9%)	<0.001
	girls	806(39.9%)	595(45.1%)	211(30.1%)	
Age(year)		12.9±2.7	12.2±2.9	14.3±1.4	<0.001
BMI(kg/m^2^)		23.8±4.8	22.0±4.0	27.3±4.2	<0.001
SBP(mmHg)		113.5±16.5	104.4±10.9	130.5±10.9	<0.001
DBP(mmHg)		62.2±18.0	54.6±15.2	76.7±13.3	<0.001
MAP(mmHg)		79.3±16.0	71.2±12.1	94.6±10.1	<0.001
PP(mmHg)		51.2±15.0	49.8±14.3	53.8±16.0	<0.001
rs9810888	TT	730(36.1%)	475(36%)	255(36.4%)	0.491
	GT	971(48.1%)	644(48.8%)	327(46.6%)	
	GG	319(15.8%)	200(15.2%)	119(17.0%)	

*p value was calculated with t-test (quantitative variables) or Chi-square test (categorical variables). SBP: systolic blood pressure. DBP: diastolic blood pressure. MAP: mean arterial pressure. PP: pulse pressure. BMI: body mass index. HBP: high blood pressure.

### Individual effect of rs9810888 on BP phenotypes

As presented in **Table 2**, under a recessive genetic model, with sex, age, age square, and study group as covariates, we found that rs9810888 was significantly associated with DBP (b = 1.78, SE = 0.74, *p* = 0.017) and MAP (b = 1.69, SE = 0.65, *p* = 0.009). With further adjustment for BMI, rs9810888 was still significantly associated with DBP (b = 1.69, SE = 0.73, *p* = 0.021) and MAP (b = 1.56, SE = 0.61, *p* = 0.010). No significant associations between rs9810888 and SBP or PP were detected (*p*>0.05). The association between rs9810888 and risk of HBP, SHBP or DHBP was also not significant (*p*>0.05, **S2 Table**).

**Table 2:** Association between the *CACNA1D* rs9810888 polymorphism and blood pressure phenotypes.

BP phenotypes	Model 1			Model 2		
	b	SE	*p*	b	SE	*p*
SBP	1.52	0.79	0.056	1.29	0.69	0.062
DBP	1.78	0.74	**0.017**	1.69	0.73	**0.021**
MAP	1.69	0.65	**0.009**	1.56	0.61	**0.010**
PP	-0.26	0.84	0.759	-0.40	0.81	0.622

Model 1 adjusted for sex, age, age square, and study group. Model 2 adjusted for sex, age, age square, study group, and BMI. SBP: systolic blood pressure. DBP: diastolic blood pressure. MAP: mean arterial pressure. PP: pulse pressure. BMI: body mass index. SE: standard error.

### Combined effects between rs9810888 and lifestyle behaviors on DBP

**Table 3**, **Fig 1(A)** and **Fig 1(B)** illustrated the associations between rs9810888 and DBP in different subgroups of lifestyle behaviors. In the participants who consumed protein (meat/fish/soy beans/egg) <twice/day, the rs9810888 GG genotype carriers had higher DBP than GT/TT genotype carriers (b = 3.78, SE = 1.66, *p* = 0.023), but in the participants who consumed protein (meat/fish/soy beans/egg) ≥twice/day, rs9810888 was not associated with DBP (*p*>0.05).

**Fig 1:**
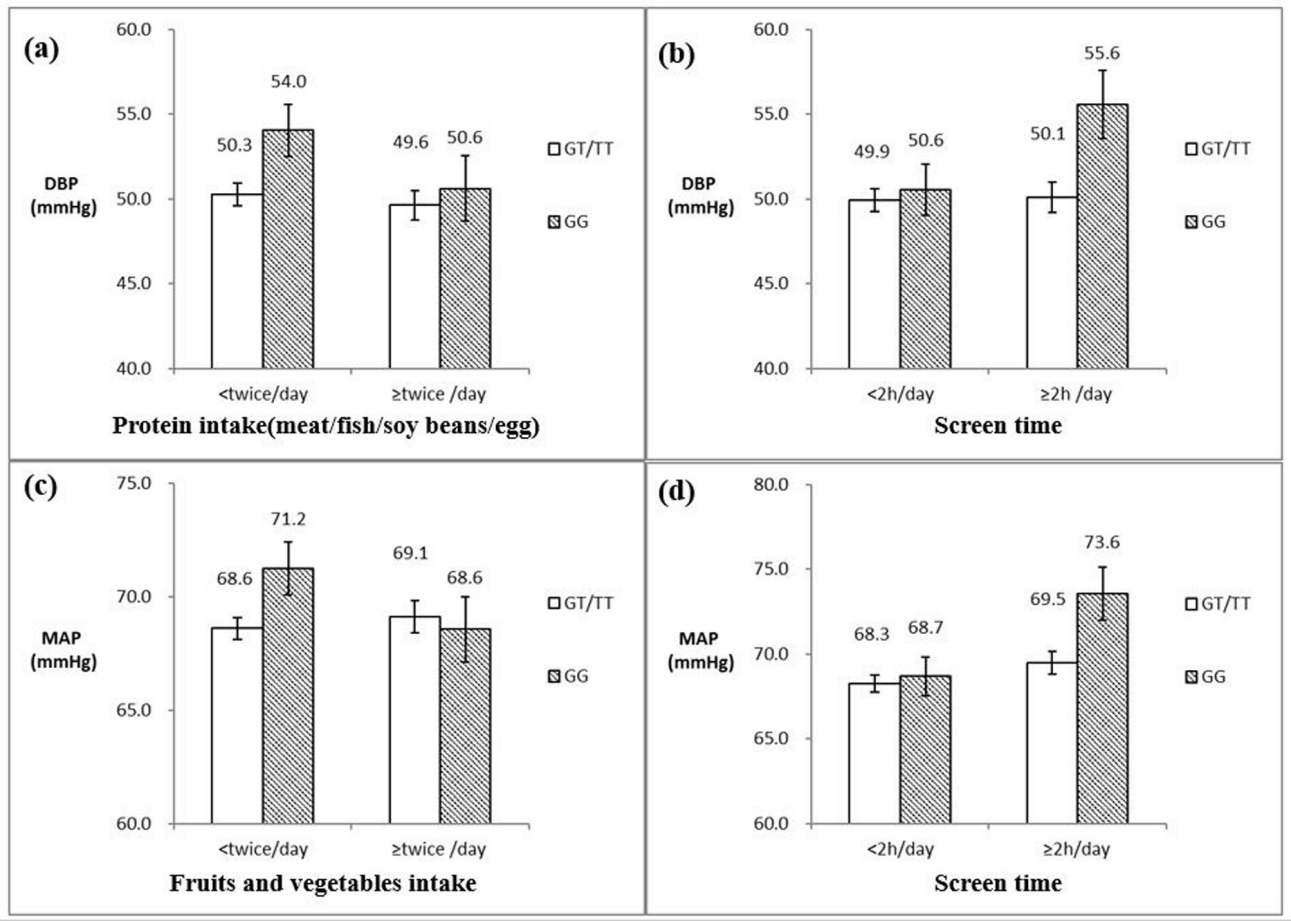
Adjusted means and standard errors of BP levels by *CACNA1D* rs9810888 polymorphism and different level of lifestyle behaviors. DBP: diastolic blood pressure. MAP: mean arterial pressure. Adjusted means and standard errors were estimated under general linear regression models with age, age square, sex and BMI adjusted. For fruit and vegetable intake, <twice/day means either fruits or vegetables intake <twice/day, and ≥twice/day means both fruits and vegetables ≥twice/day.

**Table 3:** Interaction between lifestyle behaviors and the *CACNA1D* rs9810888 polymorphism on DBP.

Lifestyle behaviors	Category	Genotype	N	Mean	SE	b	SE	*p*	*p*_interaction_
Protein intake (meat/fish/soy beans/egg)	<twice/day	GT/TT	495	50.3	0.7	3.78	1.66	**0.023**	0.295
		GG	90	54.0	1.5				
	≥twice/day	GT/TT	292	49.6	0.9	0.98	2.12	0.645	
		GG	61	50.6	1.9				
Fruits and vegetables intake^a^	<twice/day	GT/TT	583	49.8	0.6	3.16	1.65	0.055	0.257
		GG	101	52.9	1.5				
	≥twice/day	GT/TT	206	51.0	0.9	0.003	2.08	0.999	
		GG	51	51.0	1.9				
Fried chips/cakes/cookies	No	GT/TT	183	50.4	1.2	0.21	2.7	0.937	0.478
		GG	43	50.6	2.4				
	Yes	GT/TT	597	50.0	0.6	2.78	1.54	0.071	
		GG	107	52.8	1.4				
Western food	No	GT/TT	528	50.0	0.6	1.25	1.61	0.439	0.249
		GG	98	51.3	1.5				
	Yes	GT/TT	250	50.0	1.0	4.59	2.35	0.052	
		GG	51	54.6	2.1				
Soft drink	No	GT/TT	337	50.0	0.8	1.92	2.04	0.347	0.844
		GG	58	51.9	1.9				
	Yes	GT/TT	463	50.1	0.7	2.51	1.7	0.141	
		GG	95	52.6	1.6				
Physical activity	<1 hour/day	GT/TT	358	50.9	0.8	2.44	2.02	0.227	0.987
		GG	66	53.4	1.9				
	≥1 hour/day	GT/TT	429	49.2	0.7	2.38	1.75	0.173	
		GG	85	51.6	1.6				
Screen time	<2 hours/day	GT/TT	503	49.9	0.7	0.64	1.66	0.698	0.092
		GG	95	50.6	1.5				
	≥2 hours/day	GT/TT	292	50.1	0.9	5.49	2.18	**0.012**	
		GG	57	55.6	2.0				

Adjusted for sex, age, age square and BMI.

a: for fruit and vegetable intake category, <twice/day means either fruits or vegetables intake <twice/day, and ≥twice/day means both fruits and vegetables ≥twice/day.

DBP: diastolic blood pressure. BMI: body mass index. SE: standard error. The mean of BP value was adjusted for sex, age, age square and BMI.

In addition, in participants with screen time ≥2 hours/day, the rs9810888 GG genotype carriers had higher DBP than GT/TT genotype carriers (b = 5.49, SE = 2.18, *p* = 0.012), but in the participants with screen time <2 hours/day, rs9810888 was not associated with DBP (*p*>0.05).

Then we test the interaction for each lifestyle behavior and rs9810888 on DBP in general linear regression models with sex, age, age square, BMI, rs9810888, lifestyle behavior and rs9810888×lifestyle behavior as independent variables and DBP as dependent variable, but no significant interaction was identified (*p*_*interaction*_>0.05).

### Combined effects between rs9810888 and lifestyle behaviors on MAP

**Table 4**, **Fig 1(C)** and **Fig 1(D)** presented the associations between rs9810888 and MAP in different subgroups of lifestyle behaviors. In subgroups having either fruits or vegetables <twice/day, the rs9810888 GG genotype carriers had higher MAP than GT/TT genotype carriers (b = 2.64, SE = 1.27, *p* = 0.037), but in subgroups having both fruits or vegetables ≥twice/day, rs9810888 was not associated with MAP (*p*>0.05).

**Table 4:** Interaction between lifestyle behaviors and the *CACNA1D* rs9810888 polymorphism on MAP.

Lifestyle behaviors	Category	Genotype	N	Mean	SE	b	SE	*p*	*p*_interaction_
Protein intake (meat/fish/soy beans/egg)	<twice/day	GT/TT	495	68.9	0.5	2.50	1.29	0.053	0.453
		GG	90	71.4	1.2				
	≥twice/day	GT/TT	292	68.5	0.7	0.95	1.62	0.557	
		GG	61	69.4	1.5				
Fruits and vegetables intake^a^	<twice/day	GT/TT	583	68.6	0.5	2.64	1.27	**0.037**	0.132
		GG	101	71.2	1.2				
	≥twice/day	GT/TT	206	69.1	0.7	-0.57	1.60	0.724	
		GG	51	68.6	1.4				
Fried chips/cakes/cookies	No	GT/TT	183	69.6	0.9	1.27	2.00	0.524	0.892
		GG	43	70.9	1.8				
	Yes	GT/TT	597	68.5	0.5	1.70	1.20	0.157	
		GG	107	70.2	1.1				
Western food	No	GT/TT	528	68.9	0.5	1.09	1.23	0.375	0.390
		GG	98	70.0	1.1				
	Yes	GT/TT	250	68.4	0.7	3.06	1.83	0.096	
		GG	51	71.5	1.7				
Soft drink	No	GT/TT	337	68.6	0.6	0.79	1.55	0.610	0.534
		GG	58	69.4	1.4				
	Yes	GT/TT	463	68.9	0.5	2.18	1.31	0.098	
		GG	95	71.0	1.2				
Physical activity	<1hour/day	GT/TT	358	69.8	0.6	1.71	1.54	0.267	0.939
		GG	66	71.5	1.4				
	≥1hour/day	GT/TT	429	67.9	0.5	1.86	1.35	0.167	
		GG	85	69.7	1.2				
Screen time	<2 hours/day	GT/TT	503	68.3	0.5	0.42	1.26	0.738	0.094
		GG	95	68.7	1.2				
	≥2 hours/day	GT/TT	292	69.5	0.7	4.08	1.70	**0.017**	
		GG	57	73.6	1.6				

Note: Adjusted for sex, age, age square and BMI.

a: for fruit and vegetable intake category, <twice/day means either fruits or vegetables intake <twice/day, and ≥twice/day means both fruits and vegetables ≥twice/day.

MAP: mean arterial pressure. BMI: body mass index. SE: standard error. The mean of BP values was adjusted for sex, age, age square and BMI.

Additionally, in participants with screen time ≥2 hours/day, the rs9810888 GG genotype carriers had higher MAP than GT/TT genotype carriers (b = 4.80, SE = 1.70, *p* = 0.017), but in the participants with screen time <2 hours/day, rs9810888 was not associated with MAP (*p*>0.05).

Then we tested the interaction for each lifestyle behavior and rs9810888 on MAP in general linear regression models with sex, age, age square, BMI, rs9810888, lifestyle behavior and rs9810888×lifestyle behavior as independent variables and MAP as dependent variable, but no significant interaction was identified (*p*>0.05).

### Combined effects between rs9810888 and lifestyle behaviors on SBP or HBP

**S3 Table** showed the association between rs9810888 and SBP level in different subgroups of lifestyle behaviors. No combined effects or interaction was identified for rs9810888 and lifestyle behaviors on SBP level (*p*>0.05). The associations between rs9810888 and the risk of HBP in different subgroups of lifestyle behaviors were demonstrated in **S4 Table**. No combined effects or interaction was identified for rs9810888 and lifestyle behaviors on the risk of HBP (*p*>0.05).

We did sensitivity analysis to only adjust age in the results. We got similar results in the sensitivity analysis (Data not shown).

## Discussion

In the present study, we identified the individual genetic association between the *CACNA1D* rs9810888 polymorphism and DBP or MAP level among Chinese children, and there is potential difference of the genetic effect sizes across age groups (children vs adults). and for the first time we found the combined effects between *CACNA1D* and lifestyle behaviors on DBP or MAP level. We found only in the individuals who possess the unhealthy lifestyles (lower intake of protein “meat/fish/soy beans/eggs”, less fruits and vegetables intake or longer daily screen time), rs9810888 was associated with DBP or MAP level.

The *CACNA1D* rs9810888 polymorphism was firstly identified to be associated with BP among a hypertension GWAS in Chinese adults[7]. It demonstrated that rs9810888 polymorphism G allele was significantly associated with higher DBP (b = 0.39, SE = 0.06, *p* = 4.00×10^−12^). Similar to this GWAS study, we found the GG genotype of rs9810888 may be the risk genotype of higher DBP level among children in our study. The GG genotype carriers had significantly higher level of DBP than the GT/TT genotype carriers independent of BMI. But the genetic effect size of association between rs9810888 and DBP were much larger in children than adults, implying there is a potential difference across age groups.

*CACNA1D* is an important gene of calcium channel pathway and plays an important role in the regulation of the cellular calcium iron level, which is a key signal in the contraction and dilation of vascular smooth muscles[24]. As a subunit of L-type calcium channels which 4 main pore-forming α1 subunits, *CACNA1D* is widely expressed in the cells of cardiovascular system[8]. *CACNA1D* gene encodes a L-type calcium channel which could produce low-threshold, inactivating currents that resemble R-type current of the T-type current of sinoatrial node cells or many neurons, and control physiological processes, for example diastolic depolarization in these cells and neurons[25]. This could be the potential mechanism why polymorphism in CACNA1D is associated with DBP and MAP, since the neuroendocrine systems and the sinoatrial node cells plays important role in BP regulation. Nowadays calcium iron channel blocker is the first-line medication option for the treatment and control of hypertension. Kumite and colleagues demonstrated that two intron polymorphisms in *CACNA1D* (rs312481 and rs3774426, in very weak linkage disequilibrium with rs9810888, *r*^*2*^ = 0.06 and 0.07) is associated with individuals’ antihypertensive effects of L-type dihydropyridine calcium-channel blockers[26]. These findings also imply the importance of intron polymorphisms in *CACNA1D* in the BP regulation. In addition, another genetic study found several polymorphisms of *CACNA1D* were associated with aldosterone-producing adenomas and primary aldosteronism[9], which is the most common curable cause of secondary hypertension[27, 28]. Human *CACNA1D*, spanning >300kb, encodes Ca_v_1.3, which involves extensive mRNA splicing. Although rs9810888 is an intron polymorphism of *CACNA1D* gene, it may modulate the mRNA splicing of *CACNA1D* to regulate its expression. But whether or how the rs9810888 polymorphism is linked with the splicing of Ca_v_1.3’s mRNA needs further functional studies.

Furthermore, the combined effects of the *CACNA1D* rs9810888 polymorphism and lifestyle behaviors, such as screen time, protein intake (meat/fish/soy beans/egg) and fruit and vegetable intake, were found on DBP and MAP among Chinese children. To the best of our knowledge, there is no study on interaction or combined effect for *CACNA1D* polymorphism and lifestyle behaviors before. However, there are studies investigating the interaction between other genes and screen time on other phenotypes. For example, Smith and colleagues[29] found that in the subgroup with longer screen time, *LIPG* i24582 TT genotype carriers had lower high density lipoprotein cholesterol (HDL-C) than the CT/CC carriers (*p*<0.05), but the association is not significant in the subgroup with lower screen time. In an American adolescent study, Graff and colleagues also found the combined effects between obesity gene polymorphisms and longer screen time on BMI[30]. Number of studies showed that the unfavorable lifestyles, such as longer screen time, insufficient intake of fruit and vegetable, are associated with a worse BP profile[31–33]. At the same time, population genetic studies and functional studies have showed that *CACNA1D* gene is closely related with BP regulation [7, 34]. Therefore, it is highly likely that there are combined effects between these lifestyles and *CACNA1D* gene polymorphism rs9810888 on BP profile. But since the biological function of rs9810888 is still unknown now, the underlying mechanism of the combined effects between the risky lifestyles and the genetic predisposition to poorer BP profiles needs further functional studies.

Our findings of combined effects between the *CACNA1D* rs9810888 polymorphism and lifestyle behaviors on BP among children have public health implications. If the individuals who are genetically susceptible to higher BP and also have longer screen time or lower protein intake could change these unfavorable lifestyles, they may have a chance of getting the better BP profile, which has been reported to produce significant population-level change in cardiovascular diseases (CVDs) risk. According to a meta-analysis of randomized trials of antihypertensive treatment, for every 5-mmHg reduction of DBP or 10-mmHg of SBP, there would be a reduction of 22% reduction of coronary heart diseases (CHD) and 41% reduction of stroke[35].

In the present study, we analyzed the associations between rs981088 and multiple BP components (SBP, DBP, MAP, PP, HBP, SHBP and DHBP), and identified significant results in DBP and MAP. Franklin and colleagues emphasized the importance of combining multiple BP components for analysis, since combining multiple BP components was superior to predicting the CVD risk than the single BP components[36]. MAP, as an indicator of resistance, represents the average pressure during the cardiac cycle. It is more influenced by DBP according to the formula of MAP = (SBP+2×DBP)/3[18]. To some extent, the finding of associations between rs9810888 and MAP further verified results of DBP. It is reported that DBP was important indicators for CHD in younger people, while SBP was the predominant risk predictor in older subjects[37, 38]. Therefore, the individuals having the risk genotype of rs9810888 should be an important targeted population in the intervention to control the DBP and MAP level, which may also benefit for controlling the CHD risk in later life.

The present study had some strengths. Firstly, the measurement of BP was conducted by trained investigators according to a standard protocol to minimize the measurement error. Secondly, this study was conducted among children. Different from adult population, children have higher BP heritability[39, 40] and a majority of HBP children have simple HBP without other complications, which also make the pediatric population to be a more ideal population for genetic studies on BP. Thirdly, the utility of multiple BP components for analysis rather than single BP component provided more comprehensive results and insights into the genetic effect of *CACNA1D* on BP.

At the same time, there are several limitations of the present study that should be noted. Firstly, the sample size of this study provides enough power for the main effect of gene polymorphism (≥80%), but may not sufficient statistical power in interaction or combined effect analyses. Usually, interaction or combined effect studies need a relatively large sample size to produce reliable results in stratified analysis. The best method to ensure the reliability of our findings is further validation in a cohort study among children. Secondly, the behavior of physical activity could be more precisely measured by accelerometer other than a questionnaire. Thirdly, this is a case-control study, therefore the combined effects of gene and current lifestyle behaviors cannot be interpreted as causal associations. Lastly, for only one polymorphism of *CACNA1D* was studied in the present study, future studies should involve more polymorphisms.

In conclusion, our study confirmed that the *CACNA1D* rs9810888 polymorphism was significantly associated with DBP or MAP among Chinese children. In addition, we found combined effects of rs9810888 and lower protein (meat/fish/soy beans/egg) intake or longer screen time on DBP level, and also combined effects of rs9810888 and less fruit and vegetable intake or longer screen time on MAP were examined. This study is the first study investigating combined effect between the *CACNA1D* rs9810888 polymorphism and lifestyle behaviors on BP phenotypes. The findings provided new evidences for unhealthy lifestyle modification especially among children who are genetically predisposed to higher BP.

## Supporting information

S1 Table Comparison of different genetic models analyzing the associations between *CACNA1D* rs9810888 polymorphism and blood pressure level. (DOC)

S2 Table Association between the *CACNA1D* rs9810888 polymorphism and risk of HBP/SHBP/ DHBP. (DOC)

S3 Table Interaction between lifestyle behaviors and the *CACNA1D* rs9810888 polymorphism on SBP. (DOC)

S4 Table Interaction between lifestyle behaviors and the *CACNA1D* rs9810888 polymorphism on HBP. (DOC)

## Abbreviations

CACNA1D: calcium voltage-gated channel subunit alpha1 D
BP: blood pressure
BMI: body mass index
DBP: diastolic blood pressure
MAP: mean arterial blood pressure
GWAS: Genome-Wide Association Study
SBP: Systolic blood pressure
SHBP: Systolic high blood pressure
DHBP: diastolic high blood pressure
PP: pulse pressure
SD: standard deviations
MALDI-TOF MS: matrix-assisted laser desorption/ionization time-of-flight mass spectrometry
AIC: Akaike information criteria
BIC: Bayesian information criteria
