# Supplementary material for: Combined effects of the rs9810888 polymorphism in calcium voltage-gated channel subunit alpha1 D (CACNA1D) and lifestyle behaviors on blood pressure level among Chinese children
Source: PLoS One. 2019 May 30;14(5):e0216950. doi: 10.1371/journal.pone.0216950 (PMC6542524; doi:10.1371/journal.pone.0216950)
Supplement: S1 Table — (DOC) [file pone.0216950.s001.doc]

| **Supplementary Table 1. Comparison of different genetic models analyzing the associations between *CACNA1D* rs9810888 polymorphism and blood pressure level** | | | | | | |
| --- | --- | --- | --- | --- | --- | --- |
| phenotype | model | b | SE | *p* | AIC | BIC |
| SBP | Additive model | 0.28 | 0.36 | 0.440 | 9813.316 | 9852.592 |
|  | Dominant model | -0.16 | 0.53 | 0.759 | 9813.820 | 9853.096 |
|  | Recessive model | 1.29 | 0.69 | 0.062 | **9810.414** | **9849.690** |
| DBP | Additive model | 0.55 | 0.38 | 0.154 | 10027.576 | 10066.852 |
|  | Dominant model | 0.16 | 0.55 | 0.768 | 10029.531 | 10068.807 |
|  | Recessive model | 1.69 | 0.73 | 0.021 | **10024.235** | **10063.511** |
| Adjusted for sex, age, age square, study group, and BMI.SE: standard error. AIC: Akaike information criteria, BIC: Bayesian information criteria. SBP: systolic blood pressure. DBP: diastolic blood pressure. The bold font means it is the optimal model. | | | | | | |
