# Supplementary material for: Combined effects of the rs9810888 polymorphism in calcium voltage-gated channel subunit alpha1 D (CACNA1D) and lifestyle behaviors on blood pressure level among Chinese children
Source: PLoS One. 2019 May 30;14(5):e0216950. doi: 10.1371/journal.pone.0216950 (PMC6542524; doi:10.1371/journal.pone.0216950)
Supplement: S2 Table — (DOC) [file pone.0216950.s002.doc]

| **Supplementary Table 2. Association between the *CACNA1D* rs9810888 polymorphism and risk of HBP/SHBP/DHBP.** | | | | | | | | | |
| --- | --- | --- | --- | --- | --- | --- | --- | --- | --- |
| BP phenotypes | Model 1 | | | |  | Model 2 | | | |
|  | OR | 95%CI | | *p* |  | OR | 95%CI | | *p* |
| HBP | 1.20 | 0.90 | 1.61 | 0.211 |  | 1.20 | 0.88 | 1.65 | 0.249 |
| SHBP | 1.23 | 0.92 | 1.64 | 0.170 |  | 1.25 | 0.90 | 1.72 | 0.179 |
| DHBP | 1.31 | 0.93 | 1.86 | 0.124 |  | 1.29 | 0.89 | 1.86 | 0.179 |
| Model 1 adjusted for sex, age, age square, and study group. Model 2 adjusted for sex, age, age square, study group, and BMI. HBP: high blood pressure. SHBP: systolic high blood pressure. DHBP: diastolic high blood pressure. BMI: body mass index. OR: odds ratio. 95%CI: 95% confidence interval. | | | | | | | | | |
