# Supplementary material for: Combined effects of the rs9810888 polymorphism in calcium voltage-gated channel subunit alpha1 D (CACNA1D) and lifestyle behaviors on blood pressure level among Chinese children
Source: PLoS One. 2019 May 30;14(5):e0216950. doi: 10.1371/journal.pone.0216950 (PMC6542524; doi:10.1371/journal.pone.0216950)
Supplement: S3 Table — (DOC) [file pone.0216950.s003.doc]

| **Supplementary Table 3. Interaction between lifestyle behaviors and the *CACNA1D* rs9810888 polymorphism on SBP** | | | | | | | | | |
| --- | --- | --- | --- | --- | --- | --- | --- | --- | --- |
| Lifestyle behaviors | Category | Genotype | N | Mean | SE | b | SE | *p* | *p*_interaction_ |
| Protein intake (meat/fish/soy beans/egg) | <twice /day | GT/TT | 495 | 106.0 | 0.5 | -0.05 | 1.34 | 0.972 | 0.643 |
|  |  | GG | 90 | 106.0 | 1.2 |  |  |  |  |
|  | ≥twice /day | GT/TT | 292 | 106.2 | 0.6 | 0.89 | 1.56 | 0.569 |  |
|  |  | GG | 61 | 107.1 | 1.4 |  |  |  |  |
| Fruits and vegetables intake^a^ | <twice/day | GT/TT | 583 | 106.3 | 0.5 | 1.60 | 1.24 | 0.199 | 0.115 |
|  |  | GG | 101 | 107.9 | 1.1 |  |  |  |  |
|  | ≥twice/day | GT/TT | 206 | 105.4 | 0.8 | -1.70 | 1.71 | 0.320 |  |
|  |  | GG | 51 | 103.7 | 1.5 |  |  |  |  |
| Fried chips/cakes/cookies | No | GT/TT | 183 | 108.0 | 0.8 | 3.40 | 1.91 | 0.076 | 0.151 |
|  |  | GG | 43 | 111.4 | 1.7 |  |  |  |  |
|  | Yes | GT/TT | 597 | 105.4 | 0.5 | -0.47 | 1.21 | 0.698 |  |
|  |  | GG | 107 | 105.0 | 1.1 |  |  |  |  |
| Western food | No | GT/TT | 528 | 106.5 | 0.5 | 0.78 | 1.22 | 0.523 | 0.673 |
|  |  | GG | 98 | 107.3 | 1.1 |  |  |  |  |
|  | Yes | GT/TT | 250 | 105.3 | 0.8 | -0.01 | 1.88 | 0.997 |  |
|  |  | GG | 51 | 105.3 | 1.7 |  |  |  |  |
| Soft drink | No | GT/TT | 337 | 105.7 | 0.6 | -1.47 | 1.51 | 0.332 | 0.177 |
|  |  | GG | 58 | 104.3 | 1.4 |  |  |  |  |
|  | Yes | GT/TT | 463 | 106.4 | 0.5 | 1.51 | 1.33 | 0.255 |  |
|  |  | GG | 95 | 108.0 | 1.2 |  |  |  |  |
| Physical activity | <1hour/day | GT/TT | 358 | 107.4 | 0.6 | 0.25 | 1.55 | 0.874 | 0.783 |
|  |  | GG | 66 | 107.7 | 1.4 |  |  |  |  |
|  | ≥1hour/day | GT/TT | 429 | 105.1 | 0.5 | 0.83 | 1.34 | 0.537 |  |
|  |  | GG | 85 | 105.9 | 1.2 |  |  |  |  |
| Screen time | <2 hours/day | GT/TT | 503 | 105.0 | 0.5 | -0.03 | 1.22 | 0.982 | 0.537 |
|  |  | GG | 95 | 104.9 | 1.1 |  |  |  |  |
|  | ≥2 hours/day | GT/TT | 292 | 108.3 | 0.7 | 1.26 | 1.79 | 0.484 |  |
|  |  | GG | 57 | 109.5 | 1.6 |  |  |  |  |
| Note: Adjusted for sex, age, age square and BMI. a: for fruit and vegetable intake category, <twice/day means either fruits or vegetables intake <twice/day, and ≥twice/day means both fruits and vegetables ≥twice/day. SBP: systolic blood pressure. BMI: body mass index. SE: standard error. SBP: systolic blood pressure. The mean of BP values was adjusted for sex, age, age square and BMI. | | | | | | | | | |
