# Supplementary material for: Combined effects of the rs9810888 polymorphism in calcium voltage-gated channel subunit alpha1 D (CACNA1D) and lifestyle behaviors on blood pressure level among Chinese children
Source: PLoS One. 2019 May 30;14(5):e0216950. doi: 10.1371/journal.pone.0216950 (PMC6542524; doi:10.1371/journal.pone.0216950)
Supplement: S4 Table — (DOC) [file pone.0216950.s004.doc]

| **Supplementary Table 4. Interaction between lifestyle behaviors and the *CACNA1D* rs9810888 polymorphism on HBP** | | | | | | | | | | | |
| --- | --- | --- | --- | --- | --- | --- | --- | --- | --- | --- | --- |
| Lifestyle behaviors | Category | Genotype | Non-HBP | | HBP | | OR | 95%CI | | *p* | *p*_interaction_ |
|  |  |  | N | Freq(%) | N | Freq(%) |  |  |  |  |  |
| Protein intake (meat/fish/soy beans/egg) | <twice/day | GT/TT | 423 | 85.45 | 72 | 14.55 | 0.91 | 0.43 | 1.90 | 0.792 | 0.688 |
|  |  | GG | 79 | 87.78 | 11 | 12.22 |  |  |  |  |  |
|  | ≥twice/day | GT/TT | 251 | 85.96 | 41 | 14.04 | 1.19 | 0.52 | 2.68 | 0.681 |  |
|  |  | GG | 49 | 80.33 | 12 | 19.67 |  |  |  |  |  |
| Fruits and vegetables intake^a^ | <twice/day | GT/TT | 501 | 85.93 | 82 | 14.07 | 1.14 | 0.59 | 2.20 | 0.703 | 0.695 |
|  |  | GG | 86 | 85.15 | 15 | 14.85 |  |  |  |  |  |
|  | ≥twice/day | GT/TT | 177 | 85.92 | 29 | 14.08 | 0.98 | 0.37 | 2.54 | 0.960 |  |
|  |  | GG | 43 | 84.31 | 8 | 15.69 |  |  |  |  |  |
| Fried chips/cakes/cookies | No | GT/TT | 147 | 80.33 | 36 | 19.67 | 0.99 | 0.32 | 3.02 | 0.979 | 0.835 |
|  |  | GG | 38 | 88.37 | 5 | 11.63 |  |  |  |  |  |
|  | Yes | GT/TT | 521 | 87.27 | 76 | 12.73 | 1.08 | 0.58 | 2.02 | 0.800 |  |
|  |  | GG | 89 | 83.18 | 18 | 16.82 |  |  |  |  |  |
| Western food | No | GT/TT | 457 | 86.55 | 71 | 13.45 | 1.35 | 0.70 | 2.61 | 0.374 | 0.218 |
|  |  | GG | 82 | 83.67 | 16 | 16.33 |  |  |  |  |  |
|  | Yes | GT/TT | 210 | 84.00 | 40 | 16.00 | 0.67 | 0.26 | 1.77 | 0.422 |  |
|  |  | GG | 44 | 86.27 | 7 | 13.73 |  |  |  |  |  |
| Soft drink | No | GT/TT | 294 | 87.24 | 43 | 12.76 | 0.80 | 0.31 | 2.08 | 0.644 | 0.563 |
|  |  | GG | 52 | 89.66 | 6 | 10.34 |  |  |  |  |  |
|  | Yes | GT/TT | 392 | 84.67 | 71 | 15.33 | 1.16 | 0.60 | 2.25 | 0.661 |  |
|  |  | GG | 78 | 82.11 | 17 | 17.89 |  |  |  |  |  |
| Physical activity | <1hour/day | GT/TT | 302 | 84.36 | 56 | 15.64 | 0.91 | 0.38 | 2.20 | 0.837 | 0.837 |
|  |  | GG | 57 | 86.36 | 9 | 13.64 |  |  |  |  |  |
|  | ≥1hour/day | GT/TT | 372 | 86.71 | 57 | 13.29 | 1.09 | 0.55 | 2.17 | 0.811 |  |
|  |  | GG | 71 | 83.53 | 14 | 16.47 |  |  |  |  |  |
| Screen time | <2 hours/day | GT/TT | 437 | 86.88 | 66 | 13.12 | 1.01 | 0.48 | 2.15 | 0.969 | 0.984 |
|  |  | GG | 85 | 89.47 | 10 | 10.53 |  |  |  |  |  |
|  | ≥2 hours/day | GT/TT | 243 | 83.22 | 49 | 16.78 | 1.03 | 0.47 | 2.27 | 0.940 |  |
|  |  | GG | 44 | 77.19 | 13 | 22.81 |  |  |  |  |  |
| Note: Adjusted for sex, age, age square and BMI. a: for fruit and vegetable intake category, <twice/day means either fruits or vegetables intake <twice/day, and ≥twice/day means both fruits and vegetables ≥twice/day. HBP: high blood pressure. Freq: Frequency. | | | | | | | | | | | |
